# Diagnosis of vertebral fractures in children: is a simplified algorithm-based qualitative technique reliable?

**DOI:** 10.1007/s00247-015-3537-z

**Published:** 2016-02-22

**Authors:** E. Adiotomre, L. Summers, A. Allison, S. J. Walters, M. Digby, P. Broadley, I. Lang, A. C. Offiah

**Affiliations:** Radiology Department, Sheffield Teaching Hospitals NHS Foundation Trust UK, Sheffield, UK; Radiology Department, Sheffield Children’s NHS Foundation Trust, Western Bank, Sheffield, S10 2TH UK; Sheffield Medical School, University of Sheffield UK, Sheffield, UK; School of Health and Related Research, University of Sheffield UK, Sheffield, UK; Academic Unit of Child Health, University of Sheffield UK, Sheffield, UK

**Keywords:** Children, Diagnostic scoring system, Observer agreement, Osteoporosis, Radiography, Spine, Vertebral fracture, Vertebral morphometry

## Abstract

**Background:**

Identification of osteoporotic vertebral fractures allows treatment opportunity reducing future risk. There is no agreed standardised method for diagnosing paediatric vertebral fractures.

**Objective:**

To evaluate the precision of a modified adult algorithm-based qualitative (ABQ) technique, applicable to children with primary or secondary osteoporosis.

**Materials and methods:**

Three radiologists independently assessed lateral spine radiographs of 50 children with suspected reduction in bone mineral density using a modified ABQ scoring system and following simplification to include only clinically relevant parameters, a simplified ABQ score. A final consensus of all observers using simplified ABQ was performed as a reference standard for fracture characterisation. Kappa was calculated for interobserver agreement of the components of both scoring systems and intraobserver agreement of simplified ABQ based on a second read of 29 randomly selected images.

**Results:**

Interobserver Kappa for modified ABQ scoring for fracture detection, severity and shape ranged from 0.34 to 0.49 Kappa for abnormal endplate and position assessment was 0.27 to 0.38. Inter- and intraobserver Kappa for simplified ABQ scoring for fracture detection and grade ranged from 0.37 to 0.46 and 0.45 to 0.56, respectively. Inter- and intraobserver Kappa for affected endplate ranged from 0.31 to 0.41 and 0.45 to 0.51, respectively. Subjectively, observers’ felt simplified ABQ was easier and less time-consuming.

**Conclusion:**

Observer reliability of modified and simplified ABQ was similar, with slight to moderate agreement for fracture detection and grade/severity. Due to subjective preference for simplified ABQ, we suggest its use as a semi-objective measure of diagnosing paediatric vertebral fractures.

## Introduction

There is no agreed standardised method for objective diagnosis of vertebral fractures (VF) in children. In adults, there has been extensive investigation into the assessment of osteoporotic vertebral fractures but very little evaluation of techniques in children [1] for whom assessment of vertebral fractures is hindered by lack of consensus in both radiographic and morphometric definitions of fracture [2, 3]. As in adults, osteoporotic vertebral fracture identification in children is important so treatment can be commenced to reduce future fracture risk and morbidity. Currently, visual assessment, semiquantitative, morphometric and algorithm-based qualitative (ABQ) techniques are available [4]. Our aim was to identify a reliable scoring system to diagnose vertebral fractures in children, which can be applied for use in children either with primary osteoporosis such as osteogenesis imperfecta or with secondary osteoporosis such as those treated with steroids or who have leukaemia. The scoring system reflects pathological loss of vertebral body height independent of the underlying pathology.

Pure visual assessment has poor observer reliability and low reproducibility rendering it unsuitable for use in therapeutic trials or epidemiological studies [2, 5]. Automated and semi-automated quantitative computerised morphometric assessment may not adequately describe vertebral shape. Koerber et al. [6] published a semiquantitative scoring system for use in children with osteogenesis imperfecta based on three parameters (vertebral compression, thoracolumbar kyphosis and deformity type) used to attain a severity classification ranging from 1 to 5, which gives an overall assessment of the disease, or a severity score ranging from 1 to 138, which has a higher degree of differentiation that allows documentation of small changes. The system appears to be complex with the use of schematics to determine the point values of the parameters and various decision matrices to calculate the severity classification and score. It does not focus on affected endplates and a single score encompassing all vertebrae may be misleading. Genant’s semiquantitative technique is the most widely used technique in adults, developed from the radiographs of postmenopausal women. It grades vertebrae from 0 (no fracture) to 3 (severe fracture) based on an estimated percentage of vertebral height reduction and assessment of vertebral fracture shape [5] with published inter- and intraobserver reliability ranging from slight to very high [1, 7–9]. Recent published interobserver agreement using Genant’s technique in children has been moderate [1]. A review of the different scoring systems suggests that the ABQ method, which uses clear guidelines to assess changes in vertebral endplates for the identification of osteoporotic fractures regardless of height loss [4], results in the lowest estimation of vertebral fracture prevalence in adults [10]. Jiang et al. [11] found that the rigorous criteria set to exclude non-osteoporotic deformities resulted in more abnormalities being identified as non-fracture deformity or normal variants compared to qualitative and semiquantitative techniques. Severity grading of endplate depression by estimation of height reduction was subsequently added to the score [12]. The technique appears to be promising in adults to rule out non-osteoporotic fractures and non-fracture deformities [13]; however, there is only limited evaluation of the method in children. Further analysis is required to assess whether the ABQ technique is useful to identify vertebral fractures and rule out non-fracture deformities in children.

## Materials and methods

This study was completed as the side arm of a larger project (funded by the National Institute for Health Research “Research for Patient Benefit Programme” --Reference PB-PG-0110-21240) conducted in a tertiary osteogenesis imperfecta centre with the main aim to establish whether dual X-ray absorptiometry can replace spine radiographs in the assessment of paediatric vertebral morphometry thereby reducing cumulative radiation dose in children. Local Ethics Committee and Research and Development approval were granted prior to commencing the study (Reference 11/YH/0292). Informed consent/assent was obtained from all participants included in the study and accompanying responsible adults (where relevant).

Fifty consecutive patients ages 5 to 15 years were prospectively recruited between November 2011 and March 2012. Lateral spine radiographs of recruited children were obtained on one of two Phillips machines (TH3 Digital or TH Bucky Diagnost, Guildford, UK). Depending on patient size, a single thoracolumbar spine or separate thoracic and lumbar spine exposures were made. Lateral images were obtained in the lateral decubitus position. Radiation dose calculations were performed using dose area product meters preinstalled on the X-ray systems with periodic measurement of entrance surface doses to assure stability. Average dose area product was 103μGy.m^2^ (SD 99μGy.m^2^, reflecting the large size/weight and age range of 5.8 years to 15.9 years) with average exposures of 75, 84 and 74 kV for thoracic, lumbar and thoracolumbar spine radiographs respectively (detector focus distance 110 cm). Images were anonymised and read independently by three consultant radiologists (PB, IL, and ACO with 14, 13, and 10 years of experience, respectively), each with approximately 10 years of dedicated experience in paediatric radiology. Independent scores were assigned to vertebral bodies from T4 to L4. Demographic data (age, sex and diagnosis) were also collected.

Development of the scoring system was in three phases:

- Phase 1: Modification of ABQ (modified ABQ) and assessment of its reliability.
- Phase 2: Assessment of the clinical significance of the criteria used in modified ABQ.
- Phase 3: Simplification of modified ABQ (simplified ABQ) and assessment of its reliability.

### Modified ABQ

**Fig. 1:**
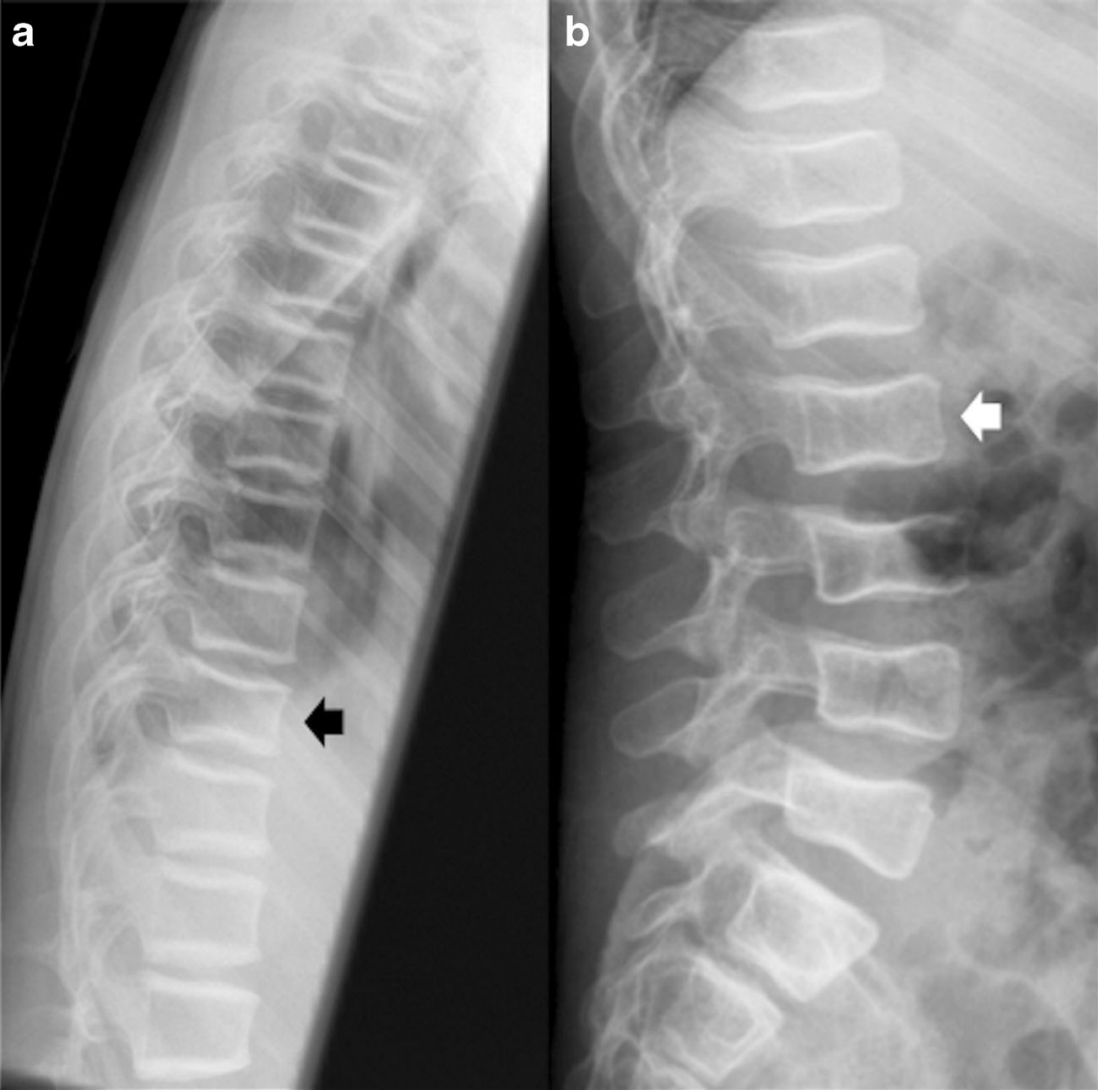
Examples of assessment using the modified and simplified scoring systems (Table 1). **a** Patient 41, a 13-year-old girl with idiopathic juvenile osteoporosis. Lateral thoracic spine radiograph illustrates the modified ABQ score. The T11 fracture (*arrow*) was independently identified by all observers as a 1aVBM fracture, which translates to a mild fracture (1a) of concave shape (V), affecting both superior and inferior endplates (**b**) in the middle of the vertebral body (M). **b** Lateral lumbar spine radiograph of Patient 32, a 6-year-old boy with atypical osteogenesis imperfect, illustrates the simplified ABQ; the L2 fracture (*arrow*) was independently identified by all observers as a 1b fracture, which translate to a height loss of less than or equal to 24% (1), affecting a single endplate (**b**). ABQ algorithm-based qualitative

A pilot of ABQ was trialed to assess its suitability and to train the observers in using the method. This consisted of 10 radiographic images read independently by the three observers followed by a consensus read. Vertebrae were subjectively assessed predominately for endplate fracture involvement using the original algorithm [4]. The original algorithm recognises Schmorl’s nodes as a non–fracture deformity as identified by focal central depression of the endplate. The endplate affected, position of endplate fracture and vertebral shape were recorded as well as estimated percentage height loss for severity grading. For example, the T11 vertebrae identified as fractured in Fig. 1 by identification of endplate disruption was graded as a 1aVBM fracture, which translates to fracture with a height loss of less than or equal to 25% (1a) of concave shape (V), affecting both superior and inferior endplates (B) in the middle of the vertebral body (M). The images used for the pilot were not used for the main study. Results were compiled by an independent researcher and distributed to observers. Discrepancies in readings were discussed and modifications to the ABQ scoring system made in order to optimise its applicability to children. After the pilot, the lateral spine radiographs of the 50 recruited patients were independently assessed and each vertebral body independently assigned a modified ABQ score by the three observers.

### Clinical survey

Following Phase 1, a short questionnaire (non-validated) consisting of seven items was developed to determine the perspective of paediatricians on the depth of detail required for the radiologic assessment of vertebral fractures in children and the clinical relevance of the criteria. The survey was sent by e-mail to 14 paediatricians, all members of the British Paediatric and Adolescent Bone Group, a specialist interest group (affiliated with the Royal College of Paediatrics and Child Health), members of which are actively involved in the clinical care of children and in research and development of new methods of investigation and treatment of children with inherited and acquired disorders of the skeleton.

### Simplified ABQ

The simplified ABQ scoring system was developed following survey results. The 50 lateral spine radiographs scored using modified ABQ were reanalysed after an interval of 3 months using the simplified ABQ scoring system, including a consensus read to act as a gold standard for fracture characterisation. Twenty-nine radiographs were randomly selected for a second read following an interval of at least 1 month, to determine intraobserver agreement of simplified ABQ.

ABQ, modified ABQ and simplified ABQ scoring systems are summarised in Table 1.

**Table 1:** Algorithm-based qualitative grading systems (ABQ)

ABQ
Grading scale:
1 = Osteoporotic fracture
2 = Non-osteoporotic short vertebral height
3 = Normal
4 = Uncertain (possible osteoporotic fracture, but uncertain due to atypical appearance or poor image quality)
5 = Unable to evaluate (poor image quality or not imaged)
Fractures classified on vertebral height reduction:
Mild ≤25%
Moderate >25% - <40%
Severe ≥40%
Modified ABQ
Grading scale:
0 = Normal
1a = Vertebral height loss ≤25% (mild #)
1b = Vertebral height loss >25%- ≤40% (moderate #)
1c = Vertebral height loss >40% (severe #)
2 = Non-osteoporotic deformity (please add comment)
3 = Uncertain or unable to determine due to quality (please add comment)
For 1a, 1b, 1c
Shape: Concave/Wedge/Crush (V/W/K)
Affected end plate: Superior/Inferior/Both (S/I/B)
Position: Anterior/Middle/ Posterior/Entire vertebral body (A/M/P/E)
Simplified ABQ
Grading scale (focusing on endplates):
Height
0 = Normal
1 = Height loss ≤ 24%
2 = Height loss ≥25%
Endplates
a = Normal
b = Single endplate affected
c = Both endplates affected
Others requiring comments
3 = Non-osteoporotic deformity
4 = Uncertain or unable to determine due to quality

Statistical analysis was performed using R software Version 3.0.2 (2013-09-25) for PC. Using the consensus simplified ABQ radiographic read as the reference standard, the prevalence of vertebral fractures was calculated as the percentage of patients identified with one or more vertebral fractures and as the percentage of fractured vertebrae from the total of 650 vertebrae assessed. Kappa statistics with corresponding 95% confidence intervals and percentages of agreement were used to assess intra- and interobserver agreement and reported as the mean agreement averaged across all 13 vertebrae for the 3 observers. Fleiss’s kappa statistic was used to assess agreement between all three observers simultaneously. Guideline values of Kappa indicated the strength of agreement as follows: poor agreement <0.20; slight 0.20 to <0.40; moderate 0.40 to <0.60, good 0.60 to <0.80 and 0.80 to 1.00 very high [14]. Numerical data is presented as suggested by Cole [15]. We did not exclude unreadable vertebrae from the analyses. For the purposes of fracture prevalence, unreadable vertebrae were analysed as not fractured. For the purposes of intra- and interobserver calculations, unreadable vertebrae were scored according to the ABQ system, for example given a score of 4 with the simplified ABQ, and agreement between readers scored accordingly. One reader may have scored a vertebra unreadable due to image quality whilst another reader may have scored the same vertebrae as fractured with a height loss of more than 25% despite the poor image quality and therefore this would be a disagreement.

## Results

The mean age of the 50 patients was 11.2 years (range: 5.8 years to 15.9 years). Fifty-six percent (28/50) were male. Forty-nine children who had suspected reduction in bone mineral density were recruited. The most common diagnosis was osteogenesis imperfecta (*n* = 39, 78%). Three children had idiopathic juvenile osteoporosis, four had unexplained fractures with or without loss of bone density and three other children had osteoporosis pseudoglioma, chronic recurrent multifocal osteomyelitis and Ehlers-Danlos syndrome, respectively. One postoperative patient with kyphoscoliosis (without suspected reduction in bone mineral density) was recruited from the spine clinic.

### Modified ABQ

Figure 1 illustrates the modified ABQ score; the T11 fracture was independently identified by all observers as a 1aVBM fracture, which translates to a mild fracture (1a) of concave shape (V), affecting both superior and inferior endplates (B) in the middle of the vertebral body (M).

Observer reliability of modified ABQ is summarised in Table 2.

**Table 2:** Summary of interobserver and intraobserver agreements

Interobserver agreement for mABQ (*n* = 50)					
			Kappa	(95% CI)	% agreement
Fracture detection (*n* = 50)	Observers	1 vs. 2	0.42	(0.37, 0.47)	76
		1 vs. 3	0.49	(0.41, 0.56)	79
		2 vs. 3	0.43	(0.36, 0.49)	78
Fracture severity (*n* = 50)	Observers	1 vs. 2	0.34	(0.30, 0.37)	71
		1 vs. 3	0.40	(0.33, 0.47)	74
		2 vs. 3	0.40	(0.33, 0.46)	76
Fracture shape (*n* = 50^a^)	Observers	1 vs. 2	0.39	(0.33, 0.45)	79
		1 vs. 3	0.45	(0.38, 0.52)	80
		2 vs. 3	0.36	(0.30, 0.42)	76
Fracture endplate (*n* = 50^a^)	Observers	1 vs. 2	0.37	(0.32, 0.42)	78
		1 vs. 3	0.37	(0.31, 0.44)	78
		2 vs. 3	0.37	(0.31, 0.42)	77
Fracture position (*n* = 50^a^)	Observers	1 vs. 2	0.38	(0.33, 0.44)	78
		1 vs. 3	0.37	(0.31, 0.43)	77
		2 vs. 3	0.27	(0.22, 0.31)	72
Interobserver agreement for sABQ (*n* = 50)					
			Kappa	(95% CI)	% agreement
Fracture detection (*n* = 50)	Observers	1 vs. 2	0.45	(0.42, 0.49)	77
		1 vs. 3	0.43	(0.36, 0.50)	74
		2 vs. 3	0.39	(0.33, 0.46)	72
	Simultaneous agreement across 3 observers (Fleiss’s kappa)		0.42	(0.37, 0.46)	62
ABQ grading (*n* = 50)	Observers	1 vs. 2	0.42	(0.37, 0.46)	74
		1 vs. 3	0.42	(0.35, 0.48)	72
		2 vs. 3	0.37	(0.31, 0.42)	69
	Simultaneous agreement across 3 observers (Fleiss’s kappa)		0.39	(0.35, 0.43)	58
Fracture endplate (*n* = 50^a^)	Observers	1 vs. 2	0.41	(0.38, 0.44)	73
		1 vs. 3	0.37	(0.31, 0.44)	69
		2 vs. 3	0.31	(0.27, 0.35)	63
	Simultaneous agreement across 3 observers (Fleiss’s kappa)		0.34	(0.31, 0.38)	55
Intraobserver agreement for sABQ (*n* = 29)					
			Kappa	(95% CI)	% agreement
Fracture detection (*n* = 29)	Observers	1	0.51	(0.39, 0.63)	82
		2	0.56	(0.49, 0.64)	82
		3	0.54	(0.45, 0.64)	76
	Average all observers		0.54	(0.48, 0.59)	80
ABQ grading (*n* = 29)	Observers	1	0.45	(0.38, 0.52)	78
		2	0.55	(0.48, 0.62)	81
		3	0.53	(0.42, 0.63)	75
	Average all observers		0.51	(0.46, 0.56)	78
Fracture endplate (*n* = 29^a^)	Observers	1	0.45	(0.38, 0.51)	78
		2	0.51	(0.44, 0.58)	79
		3	0.47	(0.38, 0.57)	71
	Average all observers		0.48	(0.43, 0.52)	76

*ABQ* algorithm-based qualitative scoring system, *mABQ* modified algorithm-based qualitative scoring system, *sABQ* simplified algorithm-based qualitative scoring system

^a^Missing values recorded as not applicable

### Clinical survey

A completed questionnaire was returned by 93% (13/14) of the paediatricians to whom they were sent. Clinicians indicated that fracture shape and endplate position were not of clinical significance and would not alter clinical management. Clinicians were most likely to initiate treatment in patients with one or more vertebral fractures with a height loss of 25% or more plus pain. Based on these results, a simple classification system for research diagnosis of vertebral fractures in children was developed as shown in Table 1.

### Simplified ABQ

Consensus read fracture characteristics:

Based on the reference standard consensus read of the 650 vertebrae assessed, 21% (137/650) were fractured. Fifty-six percent (28/50) of patients had one or more vertebral fractures and 30% (15/50) of patients had one or more vertebral fractures with a vertebral body height loss of equal to or greater than 25%. Twenty-eight percent (38/137) of the fractured vertebrae had a height loss of equal to or greater than 25%. Forty-five percent of the (61/137) fractures involved both endplates. T6 and T7 were the most fractured levels with 16 fractures at each level (23% of the total number of fractures). Twelve percent of the (77/650) vertebrae were unreadable mainly due to poor visualisation. T4 was the most unreadable level.

Figure 1 illustrates simplified ABQ; the L2 fracture was independently identified by all observers as a 1b fracture, which translates to a height loss of less than or equal to 24% (1), affecting a single endplate (b).

Table 2 summarises observer reliability and Table 3 summarises fracture characteristics for the consensus and individual reads of all observers for simplified ABQ. There was moderate simultaneous agreement among observers for fracture detection with a Fleiss’s kappa of 0.42.

**Table 3:** Summary of the simplified algorithm-based qualitative fracture score for individual observers and consensus read

	Consensus read		Observer 1		Observer 2		Observer 3	
	No.	%	No.	%	No.	%	No.	%
Total number of fractures	137	21	141	22	180	28	203	31
Most fractured level	T6 (*n* = 16), T7 (*n* = 16)		L2 (*n* = 14), L3 (*n* = 14)		L3 (*n* = 24), L4 (*n* = 24)		T6 (*n* = 27)	
Fractures involving both endplates	61	45	99	70	175	97	60	30
Fractures involving one endplate	76	55	45	32	5	3	143	70
Fractures with height loss ≤24%	99	72	95	67	151	84	178	88
Patients with ≥1 fracture	28	56	29	58	35	70	42	84
Patients with ≥1 fracture with height loss ≥25%	15	30	16	32	9	18	12	24
Patients with ≥1 fracture with both endplates affected	19	38	23	46	35	70	24	48
Total unreadable	77	12	28	4	21	3	28	4
Most unreadable level	T4 (*n* = 11)	T4 (*n* = 8)	T7 (*n* = 4)	T9 (*n* = 4)				

### Observer preference

Subjectively, observers independently felt that the simplified ABQ technique was easier to use, less time-consuming and more clinically relevant and therefore preferred using simplified ABQ compared to modified ABQ.

## Discussion

There is no agreed standardised method to diagnose vertebral fractures in children. Ideally, for clinical and research purposes, an objective, reliable and reproducible technique is required. Various techniques have been trialed in adults with Genant’s semiquantitative technique being the most common.

This is the first report to evaluate adapted versions of the ABQ technique for use in children.

Initial results showed that modified ABQ grading is useful for fracture detection, and to some extent for severity and shape analysis but not for endplate or position assessment. We concluded that further modifications were required in order to standardise readers’ observations and to note only findings of clinical relevance. Clinicians indicated that fracture shape and endplate position were not of clinical significance and would not alter clinical management. These criteria were therefore removed from the score. Clinicians were most likely to initiate treatment in patients with one or more vertebral fractures with a height loss of 25% or more plus pain. However, for research purposes, more detailed reports are required, in terms of an assessment of individual vertebral bodies, recording whether fractured or not and whether one or both endplates are affected. Therefore, based on the results of the survey, a simple classification system for vertebral fractures in children was developed to address clinical and research needs. For clinical purposes, the system reports the total number of vertebral bodies with a loss of height of less than or equal to 24% and equal to or greater than 25% and the total number of vertebral bodies with both endplates affected. For research purposes, the system reports each vertebral body as either normal, height loss of less than or equal to 24% or height loss equal to or greater than 25%, and the endplates are commented on as either normal, single endplate affected or both. The system improves the visual assessment of vertebral fractures by quantifying the severity of vertebral height loss because the paediatricians surveyed were unlikely to treat a fracture with a height loss of less than 25%. A limitation of the study is that actual measurements using workstation measurement tools only took place at the reader’s discretion. Results would have been more robust if measurements were used exclusively.

As there is no agreed standardised method of vertebral fracture diagnosis, we cannot be sure which prevalent fractures are truly fractures and as such only have the consensus radiographic read as reference standard. It was decided after the individual reads of modified ABQ and simplified ABQ to use the simplified ABQ as the reference standard for fracture characterisation with a consensus read performed by all three observers. As the objective of the study was to determine the agreement among readers and hence reproducibility, simplified ABQ was chosen as the reference standard due to the slightly higher levels of agreement. In an ideal world, a more definitive (non-ionising radiation) test such as magnetic resonance imaging (MRI), that does not rely solely on vertebral end plate disruption, shape and height loss but also takes into account marrow signal change, would be the most definitive test to identify vertebral fractures. However, clinically, MRI would be impractical given the time constraints and the number of patients seen per clinic. For research purposes, it would likely be the most appropriate test to act as a reference standard against which to compare other modalities and scoring systems. CT would be inappropriate for use in children for regular assessment of vertebral fracture risk due to high cumulative radiation doses. The larger study of which this is a side arm aims to address whether dual-energy X-ray absorptiometry could be used in place of radiographs for the diagnosis of vertebral fractures in children.

A review of the different scoring systems suggests that the ABQ method results in the lowest estimation of vertebral fracture prevalence in adults and discriminates osteoporotic vertebral fractures from non-fracture deformities [10]. Results from Halton et al. [16] from a paediatric cohort also support this with 17 out of 75 Genant semiquantitative fractures not classified as fractured, according to the ABQ algorithm. Using simplified ABQ, we found a prevalence of 21% fractured vertebrae in 56% of our patients. This is higher than a comparable paediatric study whose population was comprised of secondary osteoporosis patients [1], and a little lower than a recent paediatric osteogenesis imperfecta study with a 24% prevalence of vertebral fractures in 62% (36/58) of the patients [17]. Data from the Canadian Steroid-Induced Osteoporosis in the Pediatric Population Consortium report prevalent vertebral fracture rates using Genant’s semiquantitative technique of 16% in children with newly diagnosed acute lymphoblastic leukaemia, 7% amongst children initiating glucocorticoid therapy for rheumatic disorders and 8% in children initiating glucocorticoid therapy for nephrotic syndrome [16, 18, 19]. Clearly, fracture prevalence rate is dependent not only on the scoring system employed, but also on the population from which patients are recruited.

Twelve percent of vertebrae were classified as unreadable on the consensus read with an average of 4% classified as unreadable on the individual reads. This is similar to Siminoski et al. [1] who reported an average of 4.7% unreadable vertebrae with the upper thoracic spine having the highest percentage. Interestingly, we identified fewer fractures and more vertebrae classified as unreadable on the consensus than on the individual reads. It may be postulated that there was a higher degree of precaution taken by observers during the consensus and that fractures graded as mild on individual reads were graded as normal on the consensus and when there was uncertainty due to poor image quality more were graded unreadable. Observer 3 reported more fractures than Observers 1 and 2. Observer 3 graded a high proportion of fractures as involving only one endplate and being mild compared to Observers 1 and 2. It is postulated that the differences are due to difficulties in discriminating mild fractures involving only one endplate from subtle physiological endplate changes of normal vertebrae. In previous studies, concordance among readers and modalities has been better when mild fractures were excluded [11, 16, 20]. In our study, the majority of fractures detected (consensus read) were mild (72%), which may, in part, explain our lower Kappa values compared to those in a previous adult study, which used both the Genant and ABQ systems [11]. Furthermore, we did not exclude unreadable vertebrae from our analyses. From a clinical perspective, it is important to detect mild fractures to allow early initiation of appropriate therapy; therefore, any scoring system should aim to optimise the discrimination of mild fractures from physiological changes in vertebral morphometry.

A recent paediatric study evaluating Genant’s semi-quantitative technique from radiographs reported interobserver Kappa agreements for vertebral fracture diagnosis from 0.46 to 0.55 and intraobserver agreements from 0.53 to 0.73 [1]. The interobserver Kappa values are slightly higher than our corresponding simplified ABQ results; however, the intraobserver Kappa values are similar. In unpublished work related to a large paediatric study [21], the lead author and others have found the Genant technique to have only fair interobserver reliability. The Siminoski study had a larger cohort of 186 children; 16% (29/186) had at least one vertebral fractures, which is lower than the 56% (28/50) prevalence in our cohort [1]. A higher prevalence of completely normal or severely fractured vertebrae is likely to improve Kappa, whereas a larger number of mild fractures, which are harder to differentiate from physiological variation, is likely to result in lower Kappa values. The interobserver Kappa agreements from the first two readers in the Canadian Steroid-Induced Osteoporosis in the Pediatric Population Consortium research cohort were 0.44 for all Genant grades and 0.66 for grades 2 and 3 only [16, 19]. This is slightly higher than our Kappa results but again their prevalence of vertebral fractures was much lower. Existing scoring systems are based on vertebral body height and may be influenced by the variability in height of the developing spine. The age range of the participants in the Siminoski study was 1.3 to 17.0 years with a median age of 5.3 years compared to 11.9 years in our study [1]. As far as we are aware, no study has assessed the variability of vertebral shape in the various paediatric age groups – perhaps changes such as ossification of ring apophyses and development of Scheuermann’s disease seen in older children also account for the lower observer agreement in our study. Stratification by age may be valuable in the future analyses of results of our larger sample of 250 children.

Both Genant and ABQ are visual methods of diagnosing vertebral fractures, which are affected by the degree of observers’ experience with varying published intra- and interobserver agreements. Morphometric assessment is a quantitative computerised approach, which may potentially reduce the human factor errors from vertebral fracture diagnosis. Semiautomated models are available with a minimum of two out of six points manually positioned at vertebral boundaries in order for the software to quantify vertebral height at specified positions: anterior, middle and posterior [22]. The manual placement of points is subjective and the six points marked may not completely describe vertebral shape, misidentifying pathologies that change vertebral body shape such as Scheuermann’s disease or result in short vertebral height. Newer Active Appearance Models have been developed to use on lateral radiographs, DXA or scout CT images, which use up to 95 points to delineate the vertebral body shape either as a fully automated process with automatic vertebral search or with manual identification of the approximate vertebral centres [23, 24]. In adult lumbar spine radiographs, the accuracy of describing normal vertebrae is good but diminishes with increasing fracture grades although point-to-line accuracy below 2 mm is achieved in 79% of cases [23]. Kim et al. [24] have reported observer reliability for fracture assessment based solely on quantitative morphometric vertebral fracture definitions by a semiautomated algorithm using shape-based statistical modeling with interobserver and intraobserver Kappa of 0.67 and 0.59 to 0.69, respectively. This method may overestimate fractures as vertebral fracture definitions were actually taken from the three height ratios (anterior, middle and posterior) and not the entire vertebral body shape. DXA computer-aided vertebral fracture assessment has previously been found to be inappropriate for paediatric use due to poor image quality with numerous false-positive findings, inability to identify vertebrae in small children and failure to recognise physiological changes in morphology [25].

There are several normal variants that mimic vertebral fracture with vertebral height loss and/or endplate irregularity such as physiological thoracolumbar wedging, short vertebral height, physiological endplate interruption from indentation at anterior ring apophyses, Cupid’s bow and varying degrees of ossification that render diagnosis of (mild) vertebral fractures in children particularly difficult [26]. Theoretically, the ABQ algorithm should be able to identify true vertebral fractures by focusing on endplate disruption, but in practice this may be difficult in children given the physiological changes listed above. In contrast to adults, vertebral endplates in children are not usually parallel to each other and have an outward convex appearance because of incomplete ossification, which may limit the application of the ABQ technique in children [26]. There was persistently poorer inter-/intraobserver agreement of endplate assessment in both the modified ABQ and the simplified ABQ results compared to the other parameters. Although qualitative assessment of vertebral endplates appears useful to rule out non-fracture deformities in adults, in our study on children, it appears too subjective. A confounding factor may have been the observers’ knowledge that the radiographs were from a cohort of patients likely to have vertebral fractures; a willingness to call endplate irregularity normal physiological change may have influenced the three observers to different degrees.

In previous studies of ABQ in adults, there have only been two observers whereas this study used three experienced paediatric radiologists. Inclusion of poorly visualised vertebral bodies in the statistical analyses may be seen either as a weakness or strength of the current study. While Kappa values may have been increased had we excluded poor quality images, the data as presented demonstrates the worst case scenario and we take the pragmatic view that in these patients poor visualisation is often due to the underlying pathology (osteogenesis imperfecta) rather than to radiographic technique. Our results, therefore, demonstrate what is possible clinically. Our relatively small sample size is a weakness of the study as is the fact that we did not concurrently assess the Genant scoring system.

## Conclusion

We found moderate intraobserver and slight to moderate interobserver agreement, largely due to the discrepant values of Observer 3 compared to those of Observers 1 and 2, but no substantial difference in observer reliability of modified ABQ and simplified ABQ scores. These results were somewhat disappointing; differences among observers appear to relate to the subjective interpretation of physiological variations in shape of paediatric vertebral bodies and endplate irregularity rather than to the precise scoring system used. Subjective visual assessment can be used for routine clinical purposes but has not been advocated for use in epidemiological or therapeutic trials due to its poor reproducibility [2]. Genant’s semiquantitative assessment is the most widely used method in adults for routine clinical management, as well as epidemiological and therapeutic trials [2]. Published inter- and intraobserver reliability ranges from slight to very high [1, 7–9] and is associated with a learning curve with observer reliability improving with increased experience [2]. Although not assessed, it is likely that neither the modified nor simplified ABQ performs significantly better than standard subjective visual assessment. For clinical reporting purposes, visual subjective assessment should continue for differentiation of mild fractures from normal physiological variation. For research reporting purposes, until a scoring system is developed for children that is simple, rapid and robust, authors wishing to use a specific paediatric score should employ either the Koerber (relatively complicated) or sABQ scoring systems. In this study, all observers subjectively found simplified ABQ easier and less time-consuming, which makes it more appealing for clinical and research use compared to modified ABQ.
